# In Vivo Evaluation of the Effects of Sintering Temperature on the Optical Properties of Dental Glass-Ceramics

**DOI:** 10.3390/nano12132187

**Published:** 2022-06-25

**Authors:** Kuo-Cheng Fan, Yu-Ling Lin, Hao-Wei Tsao, Hsuan Chen, Sheng-Yang Lee, Yu-Chen Cheng, Hsiao-Ping Huang, Wei-Chun Lin

**Affiliations:** 1 Dental Department, Shin Kong Wu Ho-Su Memorial Hospital, Taipei 111, Taiwan; kelykimo@gmail.com; 2 Department of Dentistry, Wan-Fang Hospital, Taipei Medical University, Taipei 116, Taiwan; seanlee@tmu.edu.tw; 3 School of Dentistry, College of Oral Medicine, Taipei Medical University, Taipei 110, Taiwan; 4 School of Dental Technology, College of Oral Medicine, Taipei Medical University, Taipei 110, Taiwan; m249110003@tmu.edu.tw (Y.-L.L.); b210106015@tmu.edu.tw (Y.-C.C.); 5 Yue Ting Talent Smart Dental, Taipei 111, Taiwan; mysnail37@mac.com (H.-W.T.); hsuanchen@gm.ym.edu.tw (H.C.); 6 Chin Min Tai Enterprise Co., Ltd., New Taipei City 241, Taiwan; lucy-cmt@umail.hinet.net

**Keywords:** digital dentistry, dental technician, glass-ceramics, translucency parameter, dental esthetics, optical property

## Abstract

In prosthodontics, the ability of glass-ceramics to express the optical properties of natural teeth is an important goal of esthetic restorations. Dental restorations do not merely need to be similar in color to natural teeth; proper optical properties, such as opalescence, transparency, etc., must be combined in order to achieve excellent esthetic effects. The optical properties of ceramic materials are mainly distinguished by different hues (e.g., A, B, C, and D) combined with translucency (e.g., high translucency (HT), medium translucency (MT), low translucency (LT), and medium opacity (MO)). However, there are many varieties of tooth color. Therefore, it is expected that glass-ceramics can change their nanocrystal size and porosity through different heat-treatment temperatures and times and, thereby, present different transparency effects. This study mainly analyzed the influence of changes in sintering temperature on the optical properties of glass-ceramics. The optical properties of glass-ceramics in the oral cavity were evaluated with human trials. We hypothesized that (1) the transparency of glass-ceramics can be changed by controlling the sintering temperature and (2) glass-ceramics modified by the sintering temperature can be suitable for clinical applications. Results showed that the transparency decreased, the nanoparticle size increased, the crystallinity increased, and the surface hardness decreased as the sintering temperature increased. High-brightness glass-ceramics have more-sensitive optical properties. Results of clinical trials showed that glass-ceramics whose transparency was changed by controlling the sintering temperature can be candidates for clinical applications. Based on the above results, the hypotheses of this study were supported. In the future, we will continue to explore the esthetic field of dental restorations.

## 1. Introduction

In prosthodontics, dentures play a huge role in restoring oral function, comfort, appearance, and health [1]. There are many patients with different prosthesis types in the dental clinic who need a variety of dentures as oral restorations. Therefore, dental materials and manufacturing methods are continuously being improved to meet multiple clinical needs [2]. Originally, traditional restorative dentures were made of all metal. They had excellent compressive strength and wear resistance [3,4]. However, the grayish-white appearance of metal is only suitable for molars in non-esthetic areas. In addition, the oral environment is extremely complex, and alloy corrosion can easily result. Elements released from metal materials cause adverse biological reactions such as allergies and inflammation [3,4,5]. Therefore, improving the esthetics and biocompatibility of metal materials has been a major challenge in dentistry, until the emergence of dental ceramic materials to resolve the shortcomings of metal materials [6]. The advantages of ceramic materials are color stability, good biocompatibility, low plaque adhesion, high hardness, wear resistance, and the ability to exhibit natural tooth tones [7,8]. However, high brittleness, low tensile strength, and low impact resistance are the main weaknesses of ceramics [8]. In clinical practice, porcelain-fused-to-metal crowns (PFMs) are mostly made by combining the properties of metals and ceramics for dental restorations. However, PFMs are prone to ceramic fracture and staining of gingival tissues [9] and lack translucency, which limits esthetic results [10,11,12]. Therefore, materials with strength and high esthetics are still being sought for applications in dental restorations in clinical practice [13,14].

At present, patients’ requirements for esthetics are gradually increasing. Coupled with the rapid development of digital dental equipment and materials, all-ceramic restorative materials have attracted much attention [15,16]. All-ceramic materials are mainly classified into (1) zirconia ceramics and (2) glass-ceramics [17]. Zirconia is prepared using digital computer-aided design (CAD) and computer-aided manufacturing (CAM). However, the poor transparency of zirconia still needs to rely on a method of stacking ceramics to overcome the requirement of color restoration [18]. Glass-ceramics were initially produced using traditional lost wax casting and hot pressing [19,20]. The recent standardized production process of dental CAD/CAM has made the glass-ceramics process more efficient and accurate [16]. Glass-ceramics have excellent physical and chemical properties (such as excellent esthetics, translucency, low thermal conductivity, biocompatibility, chemical durability, etc.). In addition, their mechanical strength can also be improved by adding various filler particles [20,21]. At present, many manufacturers are still developing new glass-ceramics. In addition to meeting the requirements of biocompatibility and mechanical properties, the ability of glass-ceramics to express the optical properties of natural teeth meets an important goal of esthetic restorations.

Dental restorations do not just need to be similar in color to natural teeth; proper optical properties, such as opalescence, transparency, etc., must be combined in order to achieve excellent esthetic effects [10]. Opalescence occurs when light scatters at shorter wavelengths in the visible spectrum. A material has a blue appearance in reflected light and an orange appearance in transmitted light. The short-wavelength blue light in natural teeth is scattered in the enamel, giving the enamel an opalescence effect. The opalescence parameter (OP) is usually used to evaluate the performance of opalescence. The higher the OP value, the more pronounced the opalescence performance [22,23]. Transparency is the effect of regulating the surface of a material to make it transparent through light diffusion. It is one of the important factors of esthetic restorations [24]. High-transparency ceramic materials lose their ability to mask the color of the dentin. Low-transparency ceramic materials exhibit unnatural color performances [25]. Changes in translucency are usually assessed in dentistry by the contrast ratio (CR) and translucency parameter (TP). CR values range from 0 to 1, with larger values indicating greater opaqueness. CR = 0 is fully transparent and CR = 1 is fully opaque. The TP is the calculated difference between a sample placed on a black background and a white background. The numerical range of TP is 0~100. Larger values indicate higher transparency of the material [26,27]. The TP value has been used as an important criterion for selecting esthetic dental materials [28]. The optical properties of current ceramic materials are mainly distinguished by their different hues (e.g., A, B, C, and D) combined with translucency (e.g., high translucency (HT), medium translucency (MT), low translucency (LT), and medium opacity (MO)). However, there are many varieties of natural tooth colors. As a result, a variety of shades and translucency levels of ceramic materials need to be prepared in the clinic to meet the needs of each patient. Several studies showed that the composition and thickness of materials can affect the optical properties of restorations [27,29]. The glass-ceramics also show different transparency effects by changing the nanocrystal size and porosity through different heat treatment temperatures and times [30,31]. Therefore, the size of the nanoparticles added to the glass-ceramic will affect the changes in optical properties.

In this study, we mainly analyzed the influence of changes in sintering temperature on the optical properties of glass-ceramics. The optical properties of glass-ceramics in the oral cavity were evaluated with human trials. We hypothesized that (1) the transparency of glass-ceramics can be changed by controlling the sintering temperature and (2) glass-ceramics modified by the sintering temperature can be utilized for clinical applications. This research is expected to provide novel digital medical technologies in dental clinics.

## 2. Materials and Methods

### 2.1. Preparation of Glass-Ceramics

Two brands of dental glass-ceramics were used in this study (Table 1). The Amber Mill (HASS, Gyeonggi-do, Korea) groups were A3, B1, B4, and W1. Controlling the sintering temperature changed the transparency of the ceramic. For the IPS e.max CAD (Ivoclar Vivadent, Liechtenstein, Germany) group, glass-ceramics in A3 color were selected with different transparencies (HT, MT, LT, and MO). All glass-ceramics were cut with a precision low-speed cutter (CL40, Top Tech, Taichung, Taiwan) at low speed (150 rpm) with a diamond disc. Porcelain blocks were cut to thicknesses of 0.5 (tooth neck) and 1.0 mm (total crown) [32,33]. Each ceramic material and thickness was replicated 10 times. After cutting, all test pieces were ground with silicon carbide paper with different thicknesses in sequence (180/400/600/800/1200/2000). Finally, the thickness (±0.05 mm) was confirmed at five different areas on the test piece by means of Vernier calipers. The ground test pieces were crystallized in a ceramic sintering furnace (Programat CS, Ivoclar Vivadent) according to the sintering program suggested in the product specifications (Table 1). All test pieces were washed in pure deionized water using an ultrasonic oscillator to ensure the accuracy of the measurement before the color measurement.

### 2.2. Optical Image Comparison of Glass-Ceramics

Optical images of the surface of the dental glass-ceramics were assessed under a white light source, using a camera to take an optical photo to examine differences in optical properties. All samples were placed on a white background with black lines drawn to evaluate the transparency performance of the samples. In a supplementary video (Different transparency performance of the same piece of glass-ceramics.), the same tile was photographed with a digital camera, showing the performance of different transparencies. This study used ultraviolet (UV) light at a wavelength of 365 nm to irradiate the samples with natural teeth as an evaluation of the effect of opalescence.

### 2.3. Evaluation of the Optical Properties

Optical properties of the dental glass-ceramics were analyzed using a VITA Easyshade colorimeter (VITA Easyshade Compact, VITA, Zahnfabrik, Germany). Samples were placed on a background of a standard black base plate, a standard white base plate, and a VITA classical A3-shade guide (VITA classical A1–D4^®^ shade guide, VITA) for color comparisons. Five areas of all samples were separately measured to calculate an average. Finally, the color coordinates of L* (lightness), a* (red to green), and b* (yellow to blue) were obtained. The opalescence parameter (OP), contrast ratio (CR), and translucency parameter (TP) were calculated using the following equations.

#### 2.3.1. Opalescence Parameter (OP)

For the a* and b* coordinates of a test sample on a black background (indicated by B in the formula) and white background (indicated by W in the formula), we used the following formula to calculate the OP [34]:

(1) $$\mathrm{OP}=\sqrt{{\left(a_{B}^{*}-a_{W}^{*}\right)}^{2}+{\left(b_{B}^{*}-b_{W}^{*}\right)}^{2}}$$

#### 2.3.2. Contrast Ratio (CR)

The CR is the same as the TP, which mainly evaluates the change in translucency, and uses L* to calculate the Y (sharpness) coordinate value in the chromaticity space. The Y coordinate was obtained to calculate the CR value [35]:

CR = Y_B_/Y_W_; Y = [(L^*^ + 16)/116]^3^ × 100. (2)

#### 2.3.3. Translucency Parameter (TP)

The TP is a standard for measuring the difference in color transparency. The difference between the same sample placed on a black background (indicated by B in the formula) and a white background (indicated by W in the formula) was calculated by the formula [36]:

(3) $$\mathrm{TP}=\sqrt{{\left(L_{B}^{*}-L_{W}^{*}\right)}^{2}+{\left(a_{B}^{*}-a_{W}^{*}\right)}^{2}+{\left(b_{B}^{*}-b_{W}^{*}\right)}^{2}}.$$

When the TP value of a sample is equal to 0, it is completely opaque, while a sample with a TP value of 100 is completely transparent.

### 2.4. Surface Modification of Glass-Ceramics

The microstructure of the glass-ceramic surface was observed by scanning electron microscopy (SEM, Hitachi S-2400, Tokyo, Japan). Scanning electron microscopes use a high-energy electron beam to focus on the surface of a solid sample to generate a signal, which is mainly used to observe the surface texture of the material. A sample was etched for 1 min using 9% hydrofluoric acid (Porcelain Etch, Ultradent, ST, USA). It was then washed with distilled water in an ultrasonic water bath for 1 min and taken out to dry. The glass-ceramic insulating material was treated with a metal ion coating (gold plating was used for 60 s in this study). Electrons interacted with the surface of the test piece to produce a clear image.

### 2.5. X-ray Diffraction (XRD) Analysis

X-ray diffractometers are used to analyze the internal structure of materials. The transformation of the crystal phase can be understood by comparing the XRD data before and after sintering [37]. When X-rays hit the surface of a material, atoms in the material are scattered. The scattered light particles interfere with each other to form diffraction. The diffraction results can be analyzed to obtain the crystal structure. The phases of dental glass-ceramics were identified by XRD (Miniflex II, Rigaku, Tokyo, Japan). The XRD patterns were collected in the 2θ range of 10° to 50° with a scanning speed of 4°/min.

### 2.6. Surface Microhardness Test

We compared the surface hardness of glass-ceramics of different brands and transparencies before and after sintering with a Vickers hardness tester (HMV-G 20S, Shimadzu, Kyoto, Japan). The Vickers hardness tester uses a diamond indenter with a square cone with a 136° angle. It is pressed into the surface of a material for a certain time under a specific load, and the length of the diagonal of the indentation is measured after the load is removed. The hardness is calculated by the following formula [38]:

(4) $$\mathrm{HV}=\frac{F}{S}=\frac{2F\cdot \sin\left(\frac{136{}^{\circ}}{2}\right)}{g\cdot d^{2}}≅0.1891\cdot \frac{F}{d^{2}}\;\left[N/{\mathrm{mm}}^{2}\right]$$

where HV is the Vickers hardness (kgf/mm^2^), F is the indentation force (kgf), and d is the average diagonal of the indentation (mm). A force of 1.96 N for 15 s was applied to compare the effects of the surface hardness of each group of dental glass-ceramics.

### 2.7. In Vivo Evaluation of Prosthodontic Applications

The study was conducted in accord with the ethical principles of the Declaration of Helsinki. Ethical approval was granted by the Institutional Review Board (CSMUH no: CS2-20123) of Chung Shan Medical University Hospital (Taichung, Taiwan) and Yue Ting Talent Smart Dental (Taipei, Taiwan).

The human oral environment affects the esthetics of dental restorations due to saliva and the angle of light exposure. Therefore, this study obtained the consent of subjects to place anterior dental veneers in their mouths. In this way, the actual appearance of different glass-ceramics in the oral cavity could be evaluated. The study performed oral scans of patients using a CEREC AC Primescan (Dentsply Sirona, York, PA, USA). The next stage used Cerec inLab CAD/CAM (Dentsply Sirona) dental design software to design veneer patches (with a thickness of 0.5 mm for the neck and 1.0 mm for the crown). The designed files were made into veneers with a Cerec CAD/CAM dental engraving machine (MCXL, Dentsply Sirona). The veneer restorations were sintered in a ceramic sintering furnace (Programat CS, Ivoclar Vivadent) according to conditions in the instruction manual. Finally, a solid restoration sample was obtained. This study recorded optical photographs of each subject before and after wearing the veneers. Moreover, differences in color were compared by recording optical values with a dental colorimeter.

#### Color Difference (ΔE)

The color difference is also known as the “color distance”. In this study, the color difference between groups was calculated using the CIE76 color difference formula established by the International Commission on Illumination (CIE) in 1976. All samples were measured against a standard white background to obtain L*a*b* coordinates. The ΔE value was calculated using the formula below [39]:

(5) $$\Delta E=\sqrt{{\left(L^{2}-L^{1}\right)}^{2}+{\left(a^{2}-a^{1}\right)}^{2}+{\left(b^{2}-b^{1}\right)}^{2}}.$$

### 2.8. Statistical Analysis

All data are expressed as the mean ± standard deviation (SD) from 10 replicates. Data were analyzed using JMP 14 software (SAS, Cary, NC, USA). A one-way analysis of variance (ANOVA) followed by a Tukey’s honest significant difference (HSD) post hoc test was used to determine the level of significance, where *p* < 0.05 was considered significant.

## 3. Results and Discussion

### 3.1. Optical Image Comparisons of Glass-Ceramics

Human teeth have variable surface topographies [40]. The shape of a tooth affects its appearance and color. When teeth are missing, dental restorations must be made by dentists and dental technicians. Therefore, it is a major clinical challenge to make dental restorations with the same color expression as natural teeth. In this study, the heating conditions of glass-ceramic materials commonly used in dentistry were controlled to change the transparency performance of the ceramics. Transparency is affected by changing the thickness of the ceramic. Therefore, this study used common thicknesses of 0.5 (neck) and 1.0 mm (overall crown) to assess anterior restorations [32].

**Figure 1:**
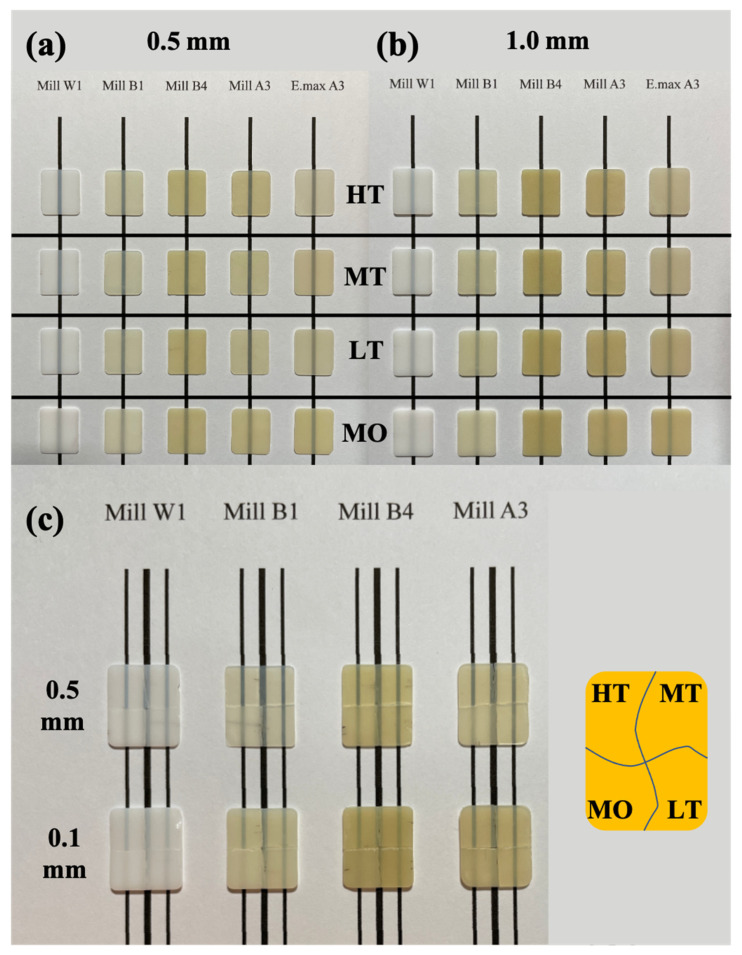
Optical photographs of dental glass-ceramics systems of various sizes, colors, and transparencies. (**a**) 0.5 mm and (**b**) 1.0 mm. (**c**) The same block was cut into four samples for sintering at different temperatures (HT 815 °C, MT 825 °C, LT 840 °C, and MO 860 °C).

Results showed that black streaks in the image could be observed from the most obvious high transparency (HT) to the least obvious (MO). This shows that these two glass-ceramics had differences in transparency (Figure 1a,b). The transparency at a thickness of 0.5 mm was higher than that at 1.0 mm. The results are similar to those in the literature, with thinner ceramic pieces having higher transparency performances [27]. This study also observed that the change in transparency was more obvious when the color of the ceramic was brighter. In the dental color classification system, W1 is the brightest followed by B1, A3, and B4. Therefore, the difference in transparency between W1 and B1 was less pronounced in optical images of this study (Figure 1a,b).

In order to confirm that the transparency of a ceramic block can change by controlling the sintering temperature, this study used the same piece of porcelain block cut into four pieces, and the seams were retained to ensure that it was the same sample. Results showed that there was a difference in transparency between the 0.5 and 1.0 mm blocks (Figure 1 and Supplementary Materials). However, the optical properties of the image still needed to be recorded by a colorimeter to accurately assess differences in color values.

**Figure 2:**
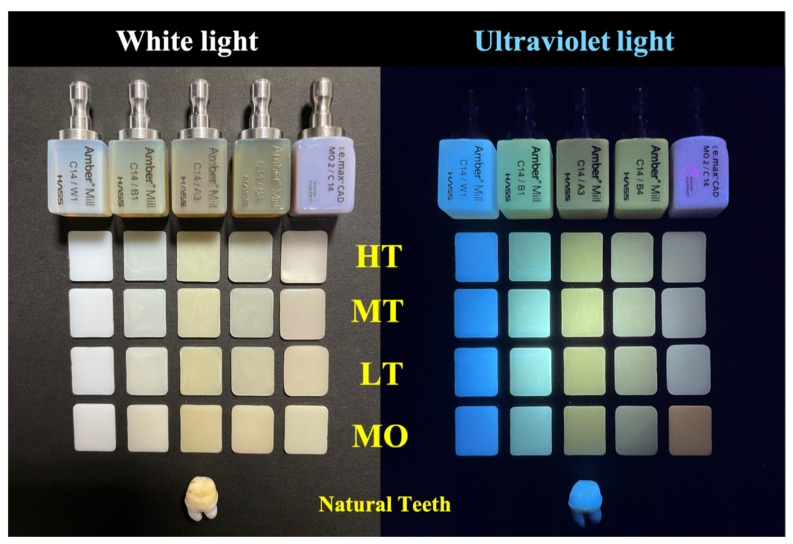
Effects of the opalescence parameter on human teeth and dental glass-ceramics under ultraviolet light (365 nm).

In addition, human teeth are composed of hydroxyapatite and collagen. There are fluorescent-like color effects on the surface, especially when exposed to UV light. Therefore, this study used both natural light and UV light to irradiate the glass-ceramics and natural teeth to evaluate the fluorescent effect. Results showed that none of the samples had a fluorescent display effect under the white light source. However, when irradiated with UV light, a fluorescent effect was exhibited (Figure 2). The W1 sample had the most similar performance to natural teeth. The fluorescence performance of other groups differed. This difference may have been caused by the addition of crystals or oxides of different colors inside the ceramic. This shows that the fluorescent effect of glass-ceramics will have different color performances due to different color systems.

### 3.2. Evaluation of Optical Properties

The human eye has certain limitations in recognizing colors and images [41]. The optical values (L*, a*, and b*) of teeth and materials can be accurately recorded using a dental colorimeter [42]. Colors can be digitized to assist in clinical dental applications. This study recorded optical values of each sample on both a black and a white background. The opalescence parameter (OP), contrast ratio (CR), and translucency parameter (TP) of the glass-ceramics were evaluated by formula calculations.

**Figure 3:**
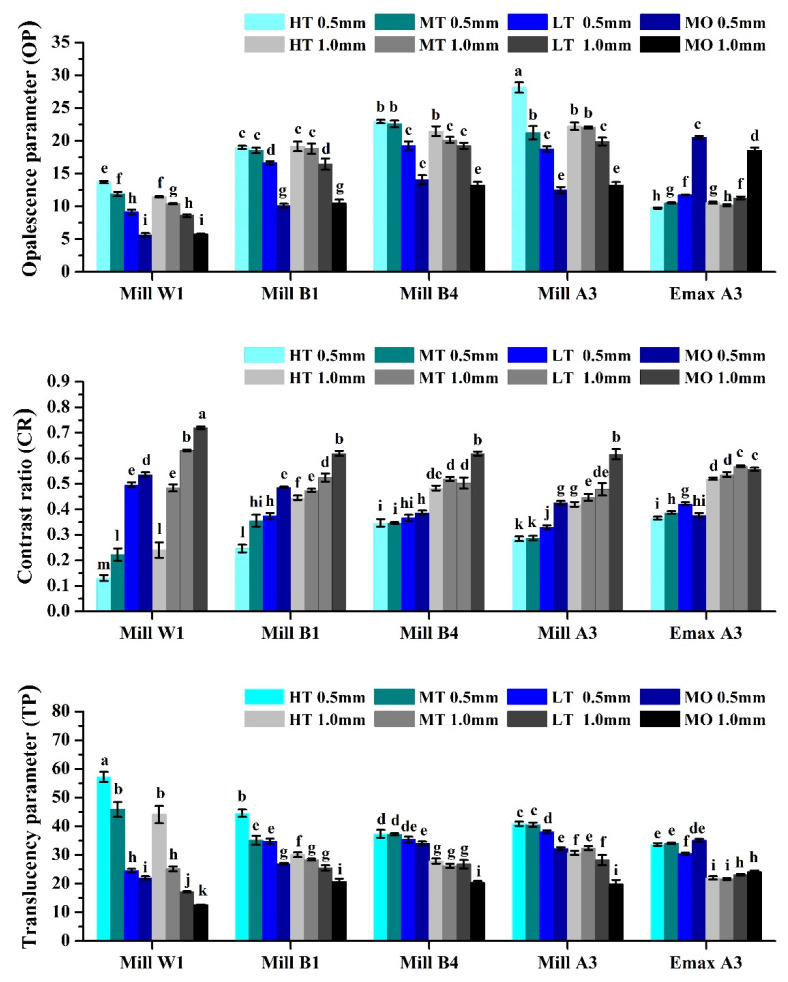
The translucency parameter, contrast ratio, and opalescence parameter of dental glass-ceramics systems. Means with different letters differ significantly (*p* < 0.05, mean ± SD, *n* = 10).

Results of the OP showed that the transparency of the glass-ceramics decreased with decreasing OP values (Figure 3). However, the OP value of the ceramics did not appear to be affected by thickness. This phenomenon is because the lightness (L*) of the sample is not considered when calculating the OP value of the sample. Therefore, there were significant differences in OP values in different color and transparency groups. Thickness did not significantly differ for OP values. Interestingly, it was found that the OP value of e.max MO was higher than those of the other groups. This shows that e.max MO is a ceramic material presenting a high OP effect. This is consistent with what the instructions said.

Contrast ratio values range from 0 to 1. A CR value of 0 is completely transparent, and a CR of 1 is completely opaque. The CR number is inversely proportional to the transparency of the material [35]. Results showed that all samples differed in transparency (Figure 3). This confirms that the effect of transparency can be changed by controlling the temperature at which the glass-ceramic is sintered. Results of CR values were similar to those in Figure 1. Brighter glass-ceramics had a positive correlation with the effect of transparency (CR value). The thickness of the ceramic significantly affected the CR value. This result supports the hypothesis of this study.

**Figure 4:**
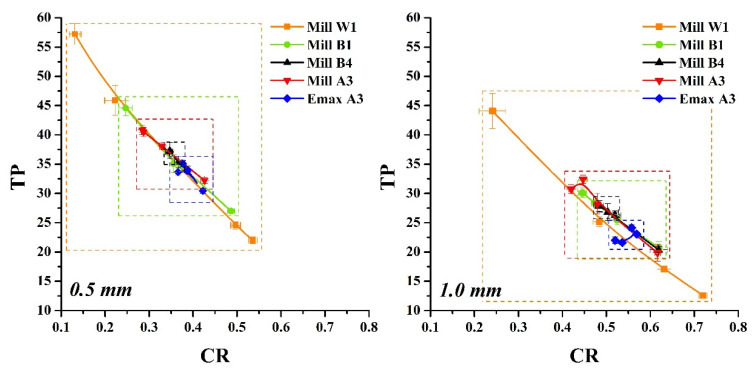
Correlation between translucency parameter and contrast ratio of dental glass-ceramic systems.

The previous literature showed that the transparency evaluation methods for dental ceramics are divided into CR and TP based ones [43]. Both can be used as indicators of the transparency of ceramic materials. The numerical results of the TP of glass-ceramics in this study were shown to be similar to those in the literature [44]. The TP value of e.max A3 HT-MO at 1.0 mm was about 20~22. Amber Mill HT-MO had a TP value of about 22~30 at 1.0 mm. The transparency of other groups was in the range 10~60 (Figure 3). Results also showed that the thickness of the glass-ceramic significantly affected the transparency performance. Moreover, this study investigated the relationship between CR and TP values (Figure 4); results showed a negative correlation between CR and TP values, which was similar to the literature [43,45]. However, we circled the range of values for glass-ceramics with different colors and transparency levels. Results showed that the W1 range of color and brightness was the largest followed by B1, A3, and B4. The range in the 0.5 mm group was significantly greater than that in the 1.0 mm group. Based on the above facts, changes in transparency of Amber Mill glass-ceramics could be achieved by controlling the sintering temperature. The brighter the color of the glass-ceramics, the more sensitive the transparency performance. Furthermore, the thickness of the glass-ceramic significantly affected the transparency performance.

### 3.3. Surface Modification of Glass-Ceramics

**Figure 5:**
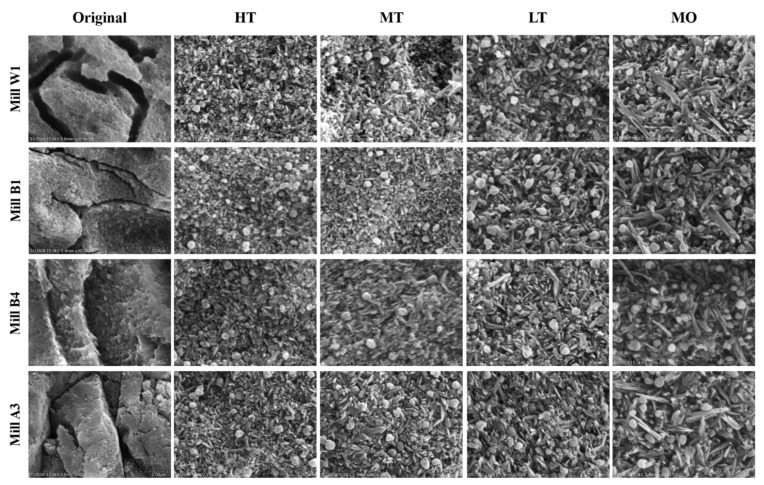
SEM images showing the microstructure of the Amber Mill ceramics at four different translucency extents and colors.

**Figure 6:**
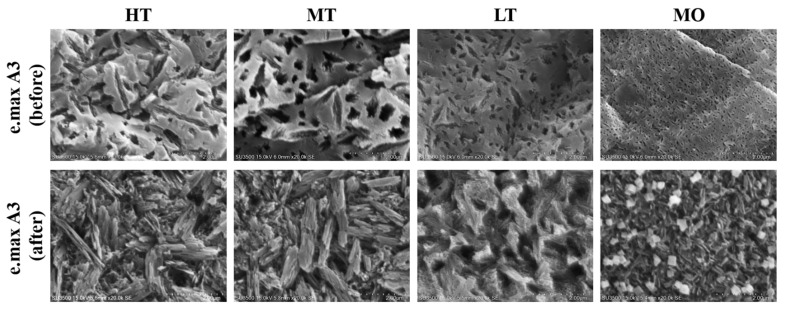
SEM images showing the microstructure of the e.max CAD ceramics in four different translucency extents.

**Figure 7:**
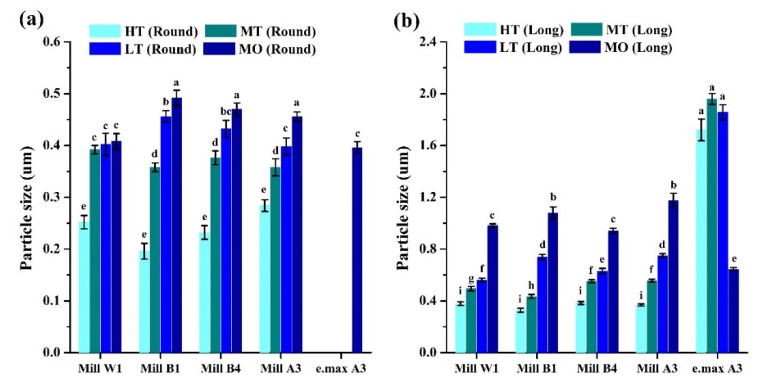
Particle sizes of different translucency extents and colors. (**a**) Round and (**b**) long. Means with different letters differ significantly (*p* < 0.05, mean ± SD, *n* = 10).

The surface microstructure of glass-ceramics was observed by SEM (Figure 5 and Figure 6). Results showed that the glass-ceramics exhibited a hollow structure before sintering [46]. After sintering, they showed round and long crystalline structures (Figure 5 and Figure 6). Crystallographic sizes of the Amber Mill and e.max groups were calculated using SEM images. Results showed that the surface granular size of the Amber Mill was 0.2~0.5 µm (Figure 7a). The size of the crystalline particles increased with increasing opacity. However, only the MO group was observed to have rounded crystals in the e.max glass-ceramics. These crystals may have been the main reason for the increased OP values of the e.max ceramic blocks.

In addition, results of long crystals found on the surface of the Amber Mill samples were similar to those of rounded crystals. The particle size was inversely proportional to the transparency (Figure 7b). However, there was no significant difference with e.max a3 HT-LT (*p* > 0.05), because e.max was applied using different ceramic blocks at the same sintering temperature. Amber Mill uses different sintering temperatures to change the transparency of the same ceramic block. The particle size also increased with an increasing sintering temperature. The literature states that high temperatures lead to high molecular mobility, low glass viscosity, and enhanced dislocations. In this way, small crystal aggregates form larger particle sizes [44]. This confirms that Amber Mill glass-ceramics had increased particle sizes at higher-temperature sintering conditions.

However, the size of long crystals was larger than the particle size of rounded crystals. In particular, the particle size of e.max a3 HT-LT specimens was about 2~4.25 times that of Amber Mill a3 HT-LT specimens.

The literature indicates that light scattering is determined by the properties of ceramics, including their impurities, pores, defects, and grain boundaries [47]. Smaller particle sizes are more likely to induce elastic scattering of the incident light wavelength, resulting in higher transparency [48]. Therefore, the particle size is one of the main factors affecting the transparency of glass-ceramics. In this study, it was observed that the transparency of the Amber Mill group was higher than that of the e.max group.

### 3.4. XRD Analysis

**Figure 8:**
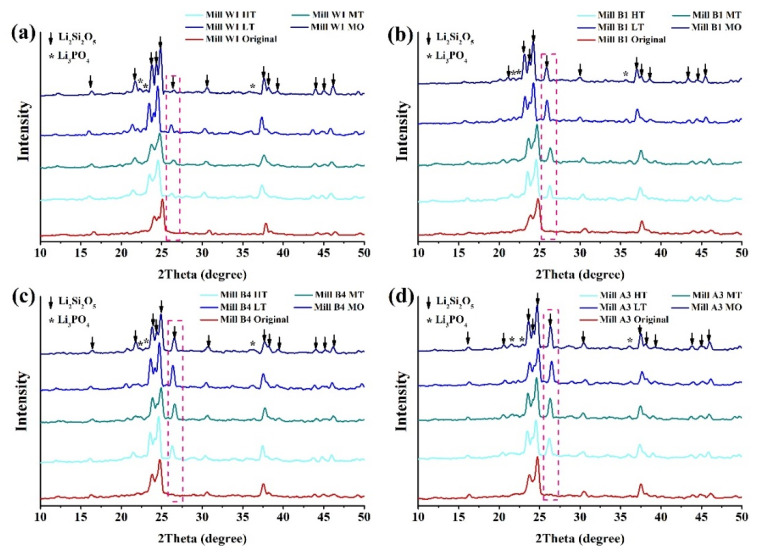
XRD representative patterns of the Amber Mill ceramics. (**a**) W1, (**b**) B1, (**c**) B4, and (**d**) A3.

**Figure 9:**
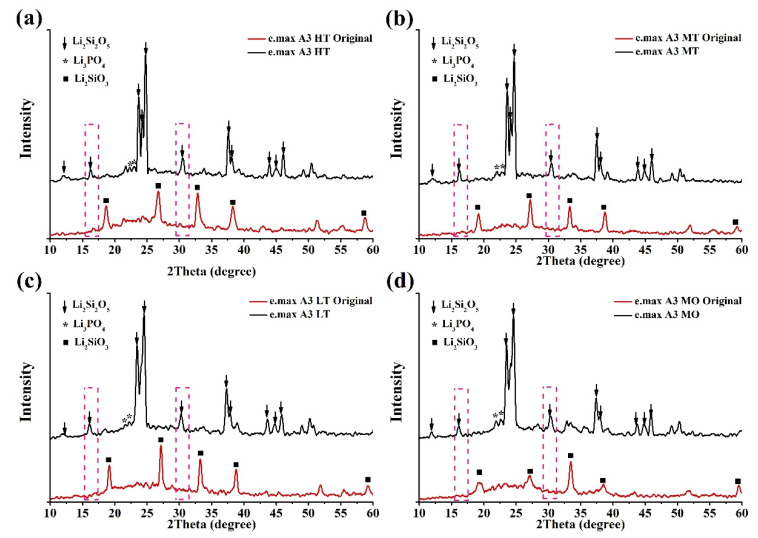
XRD representative patterns of the e.max CAD ceramics (A3). (**a**) High translucency, (**b**) medium translucency, (**c**) low translucency, and (**d**) medium opacity.

The crystal structures of glass-ceramics before and after sintering were analyzed by XRD patterns. The XRD results are shown in Figure 8 and Figure 9. Results showed that both the Amber Mill and e.max groups mainly exhibited diffraction peaks of lithium disilicate (ICCD 040-0376) [37,49]. At the same time, in all samples, Li_2_SiO_3_ before sintering was transformed to Li_2_Si_2_O_3_. The diffraction peak at 26° in the Amber Mill formation shifts was more pronounced with an increasing temperature (Figure 8). Similar phenomena occurred in other diffraction peaks. Ceramic materials increase their crystallinity at higher temperatures [37]. Therefore, diffraction peaks of MO in the Amber Mill group were higher than those of LT, MT, and HT. The results also showed that the crystalline structure of the glass-ceramics was not affected by the different colors.

In the e.max group, although the diffraction peaks were similar to those of Amber Mill, the diffraction peaks of lithium disilicate after sintering were more pronounced at 16° and 31° (Figure 9). The e.max group was made of different ceramic blocks sintered at the same temperature (850 °C). Therefore, there was almost no difference in the diffraction peak intensities at different transparency levels. In addition, it was found that the crystal structure of the glass-ceramics affected the color performance. Diffraction peaks of the Amber Mill and e.max groups significantly differed before sintering, which also caused differences in appearance (Figure 2). After sintering, it was found that the crystalline phase structures tended to be similar. This also led to the transformation of the color of the glass-ceramics to similar optical effects.

### 3.5. Surface Microhardness Test

**Figure 10:**
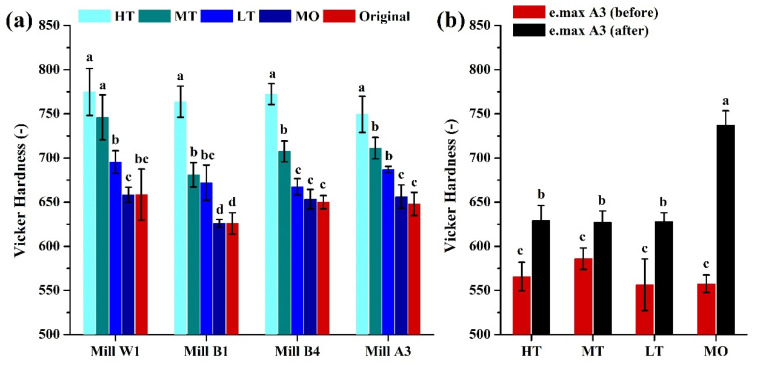
Surface microhardness of ceramic samples of different translucency extents and colors. (**a**) Amber Mill and (**b**) IPS e.max CAD. Means with different letters differ significantly (*p* < 0.05, mean ± SD, *n* = 10).

There is friction and occlusal pressure between dental restorations and natural teeth in the human mouth. Therefore, the hardness of the glass-ceramic surface was evaluated by the Vickers hardness test. Results showed that HT had the highest surface hardness in the Amber Mill group, followed by MT, LT, and MO (Figure 10a). Furthermore, there was no significant difference in surface hardness between glass-ceramics of different colors (*p* > 0.05). Results of the e.max group showed that all samples had significantly improved surface hardness after sintering (Figure 10b). There was no significant difference in the remaining groups compared to MO, which had the highest hardness.

Interestingly, the crystallinity increased as the sintering temperature increased. The sintering temperature of HT in the Amber Mill group was the lowest (815 °C), but it had the highest surface hardness. This result can possibly be explained by the particle size results. SEM images showed that the particle size of the glass-ceramic surface was inversely proportional to the sintering temperature (Figure 5, Figure 6 and Figure 7). Particles of MO in the e.max group were also the smallest, and the remaining groups showed no differences. These surface structures with a smaller particle composition can be packed more densely. Therefore, the ceramic surface would exhibit better surface hardness.

### 3.6. In Vivo Evaluation of Prosthodontic Applications

**Figure 11:**
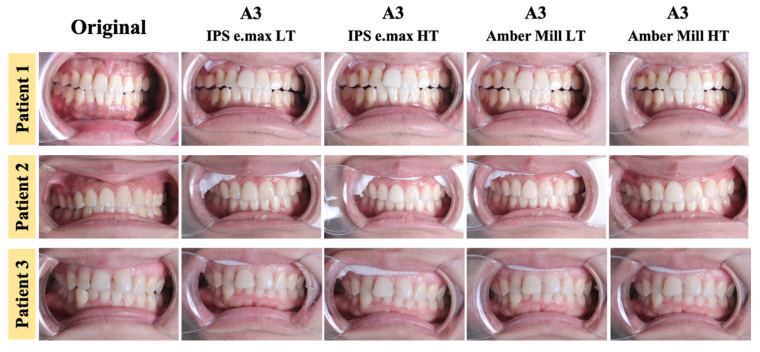
Optical images of A3 color glass-ceramics placed in the human oral cavity.

**Figure 12:**
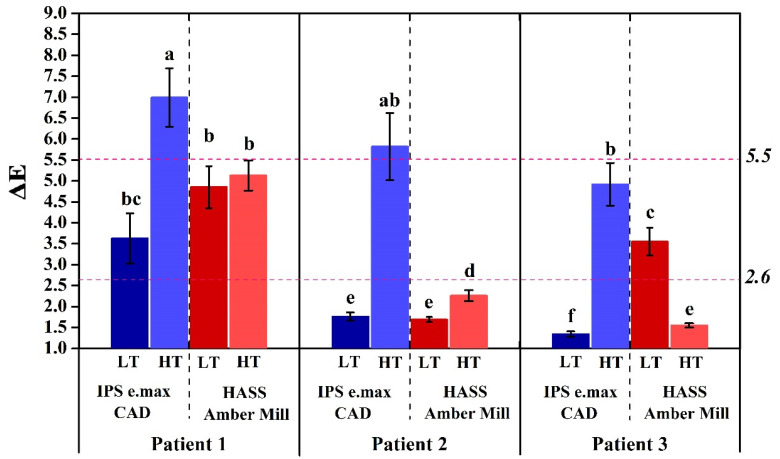
Color difference (ΔE) values of A3 color glass-ceramics placed in the human oral cavity. Means with different letters differ significantly (*p* < 0.05, mean ± SD, *n* = 3).

These days, dental clinics have to pay more attention to the esthetic effects of restorations [50,51,52]. Therefore, metal materials used in the past will gradually be replaced by all-ceramic materials [53]. In order to evaluate the actual performance of glass-ceramics in the mouth, in this study, two glass-ceramics, Amber Mill and e.max, were placed in patients’ mouths to restore anterior dental veneers. At the same time, the conditions of LT and HT transparency were used for a comparative analysis. Results showed that the HT group of e.max in oral photos had a relatively white and bright effect (Figure 11). Although the other groups were similar in color to natural teeth, the incisal part of the tooth must be personalized with staining to be closer to natural teeth. The optical values of a patient’s teeth and patches were recorded with a dental colorimeter (Table 2). Results showed that the e.max HT group presented a brighter color of A1 or B2 than A3 in the mouth of three patients. Moreover, the L* (lightness) of Amber Mill and e.max in the HT group was higher than that in the LT group. At the same time, the L* (lightness) of Amber Mill was also less than that of e.max. Therefore, both LT and HT transparency levels in Amber Mill showed a better A3 performance. The purpose of this study was to assess differences in color between glass-ceramic patches and natural teeth. Coordinated differences in optical values after patch installation were calculated using the optical properties of natural teeth as controls (Figure 12). The results in Figure 11 and Table 2 are consistent. The HT group of e.max was too bright, and the LT group of Amber Mill was too dull. Therefore, e.max HT and Amber Mill LT had the highest ΔE values among the three patients (Figure 12). The literature states that ΔE values below 2.6 are beyond the limit of the ability by the human eye to recognize them, and thus, they represent no difference. ΔE values between 2.6 and 5.5 are a clinically acceptable range [41]. Therefore, all groups could be accepted except for the e.max HT group in patients 1 and 2, which needed to be modified.

Based on the above results, the color and transparency of glass-ceramics had obvious differences due to different materials. The clinical application to dentistry requires careful evaluation of the performances of different materials. Although they were all of the same color and transparency, different types of materials had different effects.

## 4. Conclusions

This study mainly evaluated the performance of changing the transparency by controlling the sintering temperature of dental glass-ceramics. Results showed that there were effects on transparency (decreased), particle size (increased), crystallinity (increased), and surface hardness (decreased) with increasing sintering temperature. Results of clinical trials showed that glass-ceramics whose transparency can be changed by controlling the sintering temperature can be used as an alternative material for clinical applications. Based on the above results, the hypotheses of this study were supported: (1) the transparency of glass-ceramics can be changed by controlling the sintering temperature and (2) the glass-ceramics modified by the sintering temperature can be used in clinical applications. However, it is still clinically necessary for a dental technician to stain the ceramic surface. Therefore, this study was limited to the effect of the differences between nanoparticles in the original material on the optical properties. In the future, our group will continue to explore the esthetic field of dental restorations. We look forward to making helpful contributions to digital dentistry.

## Figures and Tables

**Table 1:** Description of the dental glass-ceramics systems used in the study (brand, color, transparency, sintering temperature, and size).

Sample	Translucency and Color				Size	Size
	High-Translucency Temperature (°C)	Medium-Translucency Temperature (°C)	Low-Translucency Temperature (°C)	Medium-Opacity Temperature (°C)	A	B
Amber Mill	W1	W1	W1	W1	0.5 mm	1.0 mm
	(815 °C)	(825 °C)	(840 °C)	(860 °C)		
	B1	B1	B1	B1		
	(815 °C)	(825 °C)	(840 °C)	(860 °C)		
	A3	A3	A3	A3		
	(815 °C)	(825 °C)	(840 °C)	(860 °C)		
	B4	B4	B4	B4		
	(815 °C)	(825 °C)	(840 °C)	(860 °C)		
IPS e.max CAD	A3	A3	A3	A3	0.5 mm	1.0 mm
	(850 °C)	(850 °C)	(850 °C)	(850 °C)		

**Table 2:** Optical properties of A3 color glass-ceramics placed in the human oral cavity.

Brand	Color	TP	Patient 1				Patient 2				Patient 3			
			L	a	b	#	L	a	b	#	L	a	b	#
Original tooth color			77.2	0.5	21.4	A3	75.3	0.8	22.6	A3	78.7	−0.1	19.7	A2
IPS e.max CAD	A3	LT	80.2	0.1	23.4	A3	76	1	24.2	A3	79	0.6	20.8	A3
	A3	HT	81.3	−0.3	15.8	B2	76.3	0.2	16.9	A2	80.1	−1.5	15.2	A1
HASS Amber Mill	A3	LT	74.3	−0.7	25.1	A3	75.7	−0.2	23.9	A3	76.9	0.5	22.7	A3
	A3	HT	75.6	−0.8	26.1	A3	76	−0.2	24.5	A3	77.2	−0.1	20.1	A3

TP, transparency parameter; #, colorimeter results.

## Data Availability

Not applicable.

## Supplementary Materials

The following supporting information can be downloaded at: https://www.mdpi.com/article/10.3390/nano12132187/s1, Video S1: Different transparency performance of the same piece of glass-ceramics.
